# Tumoral Phenocopies of Hypertrophic Cardiomyopathy: The Role of Cardiac Magnetic Resonance

**DOI:** 10.3390/jcm10081683

**Published:** 2021-04-14

**Authors:** Sara Bombace, Ilaria My, Marco Francone, Lorenzo Monti

**Affiliations:** 1 Department of Biomedical Sciences, Humanitas University, Via Rita Levi Montalcini 4, Pieve Emanuele, 20071 Milan, Italy; sara.bombace@humanitas.it (S.B.); ilaria.my@humanitas.it (I.M.); marco.francone@hunimed.eu (M.F.); 2 IRCCS Humanitas Research Hospital, Via Manzoni 56, Rozzano, 20089 Milan, Italy

**Keywords:** tumoral phenocopy, cardiac tumor, hypertrophic cardiomyopathy, cardiac magnetic resonance

## Abstract

Hypertrophic cardiomyopathy (HCM) is a genetic cardiac disease that presents with cardiac hypertrophy. HCM phenocopies are clinical conditions that are phenotypically undistinguishable from HCM, but with a different underlying etiology. Cardiac tumors are rare entities that can sometimes mimic HCM in their echocardiographic appearance, thus representing an example of HCM phenocopy. At present, only case reports of tumoral HCM phenocopies can be found in literature. In this systematic review, we analyzed all the published cases in which a cardiac tumor mimicked HCM to the point of misleading the diagnosis, providing a structured overview of the currently available evidence on this topic.

## 1. Introduction

Hypertrophic cardiomyopathy (HCM) is a disease that presents with thickening of the left ventricle (LV); it was first described in 1958 as a “benign cardiac tumor resulting in left ventricular outflow tract obstruction (LVOTO)” [1]. Since then, HCM has been thoroughly investigated and was eventually classified as a genetic heart disease [2], mostly due to mutations in genes encoding for sarcomeric proteins [3,4]. HCM has a high prevalence, reported to range from 1:200 to 1:500 [5]. The condition is characterized by inappropriate myocardial hypertrophy that cannot be explained by hemodynamic loading conditions alone [6]. Therefore, the diagnosis of HCM requires the exclusion of alternative etiologies of cardiac hypertrophy, including the so-called phenocopies.

HCM phenocopies are clinical conditions that present with the same phenotypic expression of HCM, but with a different etiology. They include the physiologic hypertrophic remodeling of the athlete’s heart, metabolic and storage diseases, infiltrative diseases such as amyloid cardiomyopathy, and primary and secondary cardiac tumors [7]. In particular, cardiac tumors are under recognized as HCM phenocopies, but their correct identification may significantly change the management and prognosis of the patient.

At present, only case reports can be found in literature and there are, to our knowledge, no comprehensive reviews on the oncological phenocopies of HCM. Therefore, we have collected and analyzed the case reports in which a cardiac tumor mimicked HCM to the point of misleading the diagnosis, in order to highlight any common patterns that can guide the correct diagnosis.

## 2. Methods

We performed a systematic review of the literature using PubMed for the following keywords: ‘hypertrophic cardiomyopathy’, ‘tumoral phenocopy’, ‘mimicking hypertrophic cardiomyopathy’, ‘asymmetric septal hypertrophy’, ‘cardiac tumor’, ‘cardiac metastasis’, ‘cardiac lymphoma’. We did not put any restriction criteria regarding date and original language of publication. Subsequently, we considered only publications about cardiac tumors overlapping with the diagnosis of HCM, i.e., tumoral phenocopies. All the resulting findings that matched our selection criteria were case reports.

## 3. Cardiac Tumors

Cardiac tumors are very rare entities, classified as primary and metastatic.

Based on the data of 22 large autopsy series, the frequency of primary cardiac tumors is approximately 0.02% of all autopsies [8]. Most of primary cardiac tumors are benign (75%), while the remainder are malignant. 

Metastatic tumors are malignant by definition, and occur more frequently than primary tumors, with an estimated prevalence of 1% of autopsies [9]. Any malignant neoplasm able to spread to distant sites can metastasize to the heart. Interestingly, some malignant tumors do involve the heart more often than others—for example melanoma [10]. The diagnosis of secondary cardiac metastases can occur many years after the diagnosis of the primary tumor, from which the metastatic cells originated [11].

The clinical presentation of cardiac tumors ranges from asymptomatic, with incidental findings, to severe hemodynamic impairment, with symptoms of heart failure by obstruction of the blood flow, to electrical instability with the genesis of arrhythmias, all depending on their size and location within the heart. 

Of note, a subset of cardiac tumors is associated with specific genetic syndromes (reported in Table 1), and therefore the patients may present with additional extracardiac signs and symptoms [12].

## 4. Tumoral Phenocopies of Hypertrophic Cardiomyopathy (HCM) in Literature

We have collected and reviewed the case reports in literature, in which a cardiac tumor closely mimicked HCM to the point of misleading the diagnosis. These amounted to 25 case reports, which have been illustrated in Table 2. The cases in which cardiac magnetic resonance (CMR) was eventually performed are detailed in Table 3. 

Generally, the patient would present with cardiologic symptoms and/or electrocardiographic features that could be attributable to cardiac hypertrophy. Concurrently, at echocardiography, the walls of the heart were invariably thickened, but did not plainly suggest the presence of a tumoral mass or lesion. In fact, the location and appearance of the tumoral phenocopies that most closely mimic HCM tend to overlap with the classic patterns of LV hypertrophy (LVH) due to HCM itself, i.e., at the interventricular septum and LV apex.

The tumoral phenocopy was eventually recognized only with II level imaging—most frequently with CMR—when the patient’s condition invariably worsened prompting further investigation, or incidentally during other examinations and imaging tests. 

### 4.1. Inadequate Acoustic Window

In some cases, cardiac tumors mimic HCM in their symptoms and electrocardiogram (ECG), and remain hidden from echocardiographic recognition by lingering in acoustic shadows. Torres et al. described the case of a 40-year-old male with an incidental detection of apical hypertrophy at routine ECG analysis [20]. The consequent echocardiography was inconclusive due to an inadequate ultrasound window. Eventually, CMR was needed to detect the mass at the apex of the LV, and it also allowed the diagnosis of cardiac fibroma: the mass was isointense compared to the muscle on T1 weighted images, and hypointense on the first pass study, whilst being strongly enhanced on delayed imaging, on contrast-enhanced T2 weighted images.

### 4.2. Adequate Acoustic Window

Echocardiography may miss the correct diagnosis even in patients with an adequate ultrasound window. Hovasse et al. presented a case of a 50-year-old male with exertional dyspnea and a systolic murmur [17]. Transthoracic echocardiography initially pointed to a diagnosis of obstructive HCM (OHCM) with systolic anterior motion (SAM) of the anterior mitral leaflet, without further suspicion of any alternative diagnosis. Nonetheless, the patient underwent CMR for risk stratification and assessment of fibrosis. At CMR the presence of a lobular mass was detected, and this prompted a correction of the diagnosis to that of benign myocardial tumor. Furthermore, through tissue characteristics analysis, the type of tumor could be determined as a rhabdomyoma, displaying the same T1 weighted and T2 weighted signals as the normal myocardium.

Similarly, in a case presented by Papadopoulos et al., a 46-year-old male underwent echocardiographic imaging which appeared suggestive of asymmetrical LVH [26]. However, further investigation with CMR, originally aimed at the evaluation of myocardial fibrosis, showed extensive adipose infiltration of the interventricular septum in a heterogeneous pattern and not well-demarcated, highly compatible with the diagnosis of lipomatous hypertrophy of the interventricular septum instead.

### 4.3. Apical HCM

In case of apical left ventricular masses, the differential diagnosis with apical HCM (AHCM) is particularly challenging with echocardiography, because of the variable presentation of AHCM, and because both instances are characterized by a diffuse thickening of the LV apex. Veinot et al. reported a case of a 55-year-old male with a cardiac fibroma, who carried the diagnosis of AHCM for more than 10 years [21]. He never underwent level II imaging, i.e., CMR, for example. At echocardiographic reassessment, performed due to worsening of the symptoms, a large mass involving the lateral and apical left ventricular walls with areas of calcification was revealed that had never been noticed in previous exams. A subsequent CMR confirmed the presence of the calcified mass, with no contrast enhancement, and strongly suggested the diagnosis of a cardiac fibroma. Surgical excision of the tumor was performed, and the diagnosis was confirmed. 

### 4.4. Malignant Phenocopies Red Flags

#### 4.4.1. Echocardiographic Red Flags

**Figure 1:**
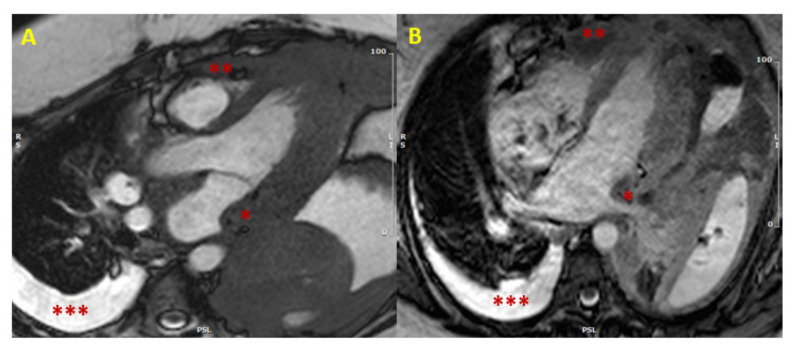
Cardiac lymphoma at CMR. SSFP images in long axis (3-chamber view in Panel (**A**), and 4-chamber view in Panel (**B**)) showing concomitant hypertrophy of left atrial wall (single asterisk *) and RV (two asterisks **). The presence of multiple areas of hypertrophy and pleural effusion (three asterisks ***) are “red flags” for malignancy. (CMR = cardiac magnetic resonance; SSFP = steady-state free precession; LV = left ventricle; RV = right ventricle).

In case of hypertrophy of the interventricular septum at echocardiography, some features may be considered as red flags: among these are a concomitant thickening of the right ventricle, of the atrial walls or of the interatrial septum, and pericardial and/or pleural effusion, because they imply a higher probability of malignancy, and make the diagnosis of a common HCM less likely (Figure 1). Representative of this is the case reported by Kuchynka et al. of a 38-year-old male, with a month-long history of abdominal lymphadenopathy, whose echocardiographic imaging at workup gave grounds for suspicion of HCM [29]. Upon renewed ultrasound investigation, the thickening of the interventricular septum was confirmed, but also a thickening of the right ventricle’s free wall and of the interatrial septum, with a large pericardial effusion, became evident. The ensuing CMR showed diffuse heterogenous late gadolinium enhancement (LGE) in the thickened segments of the myocardium, highly suggestive of a lymphoproliferative disease. Eventually, histological analysis of an enlarged lymph node confirmed the diagnosis of non-Hodgkin lymphoma. After chemotherapy, complete remission was achieved, with normalization of ventricular wall thickness and resorption of the pericardial effusion. 

#### 4.4.2. Clinical Red Flags

A prior clinical history of malignancy should be considered as a red flag, too. In fact, secondary cardiac metastases can occur many years after the diagnosis of the primary tumor and, therefore, any recent diagnosis of HCM in a patient with a prior history of malignancy should be carefully investigated, in order to exclude a metastatic phenocopy of HCM: to this end, CMR serves as an excellent, unrivaled tool. This was evident in the case of a 61-year-old hepatocellular carcinoma (HCC) patient treated with radiofrequency ablation of the liver lesion, and a precedent diagnosis of HCM, presented by Greco et al. [36]. After a syncope-like episode, echocardiographic re-evaluation detected a large isoechogenic ventricular septum size (approximately 5.58 cm), extending to the cardiac apex. Concomitantly, at body computed tomography (CT) scan, a distant recurrence of the previously treated nodular lesion was found in the liver. A final CMR unmasked the lesion in the heart as causative of the abnormal thickness of the interventricular septum. The lesion displayed tenuous polylobate gadolinium enhancement and caused significant restriction of the outflow of left and right ventricles during systole. A myocardial biopsy eventually confirmed the infiltration of a well-differentiated HCC metastasis in the ventricular septum.

Since some cardiac tumors can be associated to genetic syndromes, a complete and thorough physical examination is necessary in every patient with a new diagnosis of HCM, in order to exclude syndromic traits. The presence of any signs and symptoms attributable to genetic syndromes such as tuberous sclerosis, Gorlin syndrome and Carney complex, imposes the need of a II level evaluation, in order to exclude an oncologic phenocopy of HCM. Pawloska et al. reported a case of a 41-year-old male patient with a longstanding history of HCM, that was treated with the positioning of an implanted cardioverter-defibrillator (ICD), seven years before [22]. None of the multiple precedent transthoracic echocardiographic examinations ever described the presence of a cardiac mass, or suggested an alternative diagnosis. Only a transesophageal echocardiography, eventually performed for the evaluation of atrial and ventricular lead failure of the ICD, and a subsequent chest CT, revealed the presence of a cardiac fibroma; of note, the ICD precluded the execution of CMR in this patient. The chest CT also displayed a bifid third rib, and therefore Gorlin syndrome was suspected. A more accurate history taking, and a meticulous physical examination ascertained other findings, such as a history of multiple basal cell carcinomas, macrocephaly, and plantar pitting. In conclusion, the final diagnosis of Gorlin syndrome was warranted. 

## 5. Imaging of Tumoral Phenocopies

### 5.1. Echocardiography

Some cardiac tumors may closely mimic HCM in their echocardiographic appearance. At ultrasound imaging, a diagnosis of HCM can be made when the maximal end-diastolic wall thickness is >15 mm anywhere in the LV, in the absence of other causes of LV hypertrophy (LVH) [6,42].

In HCM nearly any part of the ventricular wall may become hypertrophic [42,43]. The classical phenotype of HCM, however, presents with asymmetric LVH of the basal anterior septum, displaying hypertrophy of the basal interventricular anterior septum in continuity with the anterior free wall. Albeit less commonly, HCM may as well cause LVH with non-septal asymmetric patterns, as well as with symmetric and concentric patterns [43]. Of note, apical HCM (AHCM), classically known as Yamaguchi syndrome, is a specific and rare variant of HCM with the isolated thickening of the LV apex [44].

Some secondary ancillary findings are typically associated with HCM at echocardiography, although they are not required for the diagnosis: these include hypertrophic and apically displaced papillary muscles, an anomalous insertion of the papillary muscle directly at the anterior leaflet of the mitral valve in the absence of chordae tendinae, elongated mitral valve leaflets, and myocardial clefts [42]. 

Benign cardiac tumors can range from pedunculated to sessile masses, protruding or not at various degrees into the cardiac chambers. These masses can be clearly visible at ultrasound imaging, but an incorrect diagnosis of HCM can be made when the tumor is not morphologically well-demarcated, when the location and appearance overlaps with the classic HCM patterns of LVH, or when it hides in areas of acoustic shadow. 

Lymphomas may mimic HCM very well, too, because they appear as diffuse thickening of the cardiac walls without any apparent intracardiac mass, and thus evade echocardiographic recognition with ease. However, the presence of pericardial effusion, which often accompanies the myocardial thickening in these patients, suggests the possibility of a malignancy, rather than HCM [29,30,31,32,33,34,35].

On the contrary, there seem to be no cases in literature, in which cardiac sarcomas are misdiagnosed as HCM at echocardiography. Cardiac sarcomas usually arise from either of the atria, or from the pericardium [45] and present as an intracavitary mass [46] or as recurrent pericardial effusion [47]; their distinct manifestation usually allows echocardiography to be effective for the initial identification of the lesion. 

Cardiac metastases involving the myocardium can often present with the same phenotype of HCM, displaying thickening of myocardial walls or mass formation, which can be misinterpreted as HCM [11].

### 5.2. Second-Level Imaging

With the use of CMR, the distinction between HCM and its tumoral phenocopies at workup is significantly improved, due to the ability of CMR in soft tissue characterization [48].

CMR is well recognized as the imaging modality of choice for the detection and evaluation of cardiac tumors [45,49,50,51]. A comprehensive study for the characterization of areas of myocardial hypertrophy should include T1 and T2 short-tau inversion recovery (STIR) fat-suppressed images, parametric sequences for native T1 and extracellular volume (ECV) calculation, first pass perfusion imaging and LGE sequences. CMR gives detailed information about tumor location, dimension, morphology and hemodynamic effect, about tissue composition (fat–cellularity–fibrosis) and perfusion, and about infiltration of the surrounding tissues. Of note, microcalcifications may be missed at CMR. Parametric sequences are of paramount interest and are still underutilized in the setting of oncologic phenocopies of HCM: typically, HCM shows increased native T1 and ECV values. In the case of cardiac tumors, ECV calculation often show normal/reduced values (of 25% or less) since the hypertrophic myocardium is characterized by tumor hypercellularity rather than extracellular volume expansion due to fiber disarray [52].

Moreover, some cardiac tumors display a specific CMR signal, which make a confident diagnosis possible, even without the use of contrast media. For example, cardiac metastases of melanomas are typically hyperintense in T1-weighted and hypointense in T2-weighted pre-contrast images. 

However, in some cardiac tumors, such as Hamartoma of mature cardiac myocytes, CMR fails to detect the presence of a mass [18,19].

In these cases, CMR can be complemented by other imaging techniques: these include PET-CT and 18-FDG PET [30]. The latter can be particularly advantageous in this clinical setting, documenting the presence or absence of metabolically active tissue [53]. These techniques require a consistent suspicion of a tumor to be executed, that can be strengthened by the CMR evidence of a normal conventional and T1–T2 mapping features in non hypertrophic segments. 

**Figure 2:**
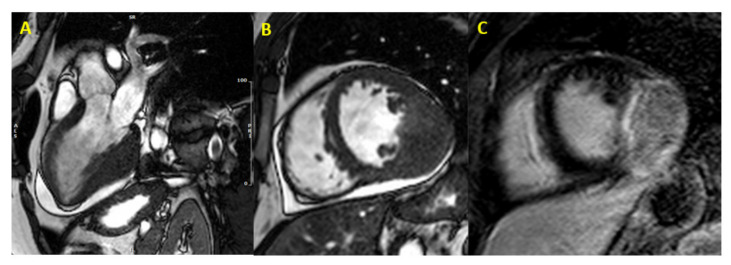
Cardiac fibroma at CMR. Focal myocardial thickening of mid-basal infero-lateral wall, with isointense signal in basal SSFP sequences, mimicking HCM (Panel (**A**,**B**)). Heterogeneous LGE with a capsulated appearance of the focal hypertrophy suggesting cardiac fibroma (Panel (**C**)). (CMR = cardiac magnetic resonance; SSFP = steady-state free precession; HCM = hypertrophic cardiomyopathy; LGE = late gadolinium enhancement).

**Figure 3:**
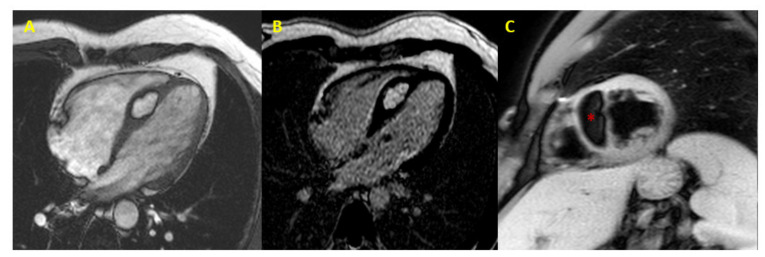
Cardiac lipoma at CMR. CMR image (4-chamber view) showing septal hypertrophy characterized by hyperintense signal in SSFP and LGE sequences (Panel (**A**,**B**)), and signal suppression (* asterisk in Panel (**C**)) in fat-saturated images (CMR = cardiac magnetic resonance; SSFP = steady-state free precession; LGE = late gadolinium enhancement).

Figure 1, Figure 2 and Figure 3 provide some examples of cardiac tumors detected by CMR.

Table 2 comprises the case reports of tumoral phenocopies and illustrates their typical features [9,11,49,54].

## 6. Discussion

By reviewing the published reports on tumoral phenocopies of HCM, we have attempted to highlight the diagnostic challenges associated with this condition.

A correct diagnosis is cardinal; the suspicion of an oncologic phenocopy of HCM may lead to a completely different diagnostic workup, treatment and prognosis.

When the surgical removal of a cardiac tumor is possible, it may lead to an improved outcome for the patient. Similarly, hematologic, and generally malignant tumors must be recognized in order to promptly initiate a correct chemotherapeutic regimen, with variable results depending on the tumor, but with the possibility of a good prognosis and even complete regression at follow up, such as in the case of many lymphomas. 

Regarding prognostic stratification, the sudden cardiac death (SCD) risk prediction model promoted by the European Society of Cardiology (ESC) for HCM cannot be applied for the tumoral phenocopies [6], whose risk stratification depends on different variables.

Moreover, recognizing an HCM tumoral phenocopy permits to avoid wasting of clinical resources on the wrong diagnosis and its consequent management: for example HCM warrants for genetic testing of the patient and screening of their relatives, whereas the tumoral phenocopies do not [42]. 

Current ESC and American Heart Association/American College of Cardiology (AHA/ACC) guidelines for HCM consider echocardiography pivotal to the diagnosis of HCM [6,42], and give a class I indication to CMR in case of inconclusive echocardiographic imaging or the suspicion of an alternative diagnosis. Notably, the ESC and AHA/ACC guidelines recognize the ability of CMR to give additional information for arrhythmic risk stratification. Consequently, many HCM patients may never undergo CMR during the clinical course of their disease. On the other hand, the 2020 consensus paper by the Society for Cardiovascular Magnetic Resonance (SCMR), gives a class I indication to CMR for all HCM patients [48]. 

The limited number of case reports of tumoral phenocopies of HCM in the literature and their variable etiology do not allow for a formal recommendation on the systematic use of CMR in all HCM patients. 

In our opinion, CMR should be considered in all suspected HCM patients who have a relevant tumoral history or additional extracardiac signs and symptoms: the former may conceal a cardiac metastasis from an extracardiac tumor, while the latter may have a genetic syndrome that involve cardiac tumors. 

Of interest, tumoral phenocopies of HCM never show the ancillary echocardiographic findings often associated with typical HCM; the SAM of the mitral valve and LVOTO have been described [17,23,25,31], but they represent the hemodynamic consequence of septal hypertrophy, rather than a specific morphological feature of HCM. CMR should, therefore, be considered in all HCM patients presenting with the echocardiographic red flags mentioned in Table 4, including the absence of ancillary findings of HCM. These red flags, even if not onco-specific, may be considered suspicious for a possible phenocopy, including other cardiomyopathies such as amyloidosis, Fabry’s disease that may also be associated with these imaging features [55,56]. The diagnosis of tumoral phenocopies of HCM somehow represents an unmet medical need, and CMR displays an enormous diagnostic potential in this regard.

## 7. Conclusions

We collected published cases in which echocardiography was unable to distinguish HCM from its rare tumoral phenocopies. From our literature review, CMR emerged as a powerful II-level diagnostic tool in order to refine these challenging diagnoses.

We identified a series of clinical and echocardiographic features, or “red flags”, that characterize the published series of tumoral phenocopies. Therefore, we suggest keeping these red flags in mind in any new diagnosis of HCM at echocardiography. If they are considered during diagnostic workup, they may help to raise clinical suspicions (and further CMR evaluation) about the presence of an oncologic phenocopy of HCM.

## Figures and Tables

**Table 1:** Main genetic syndromes associated to cardiac tumors.

Syndrome	Trasmission	Mutation	Cardiac Tumor	Frequency	Extracardiac Signs and Symptoms	Reference
Tuberous Sclerosis	Autosomal dominant	TSC1 or TSC2	Rhabdomyoma	80%	Hamartomas in several organs, including heart rhabdomyomas and hamartomas of brain, skin, eyes, kidney, lung, and liver	[13,14]
Gorlin Syndrome	Autosomal dominant	PTCH1	Cardiac Fibroma	3%	Predisposition to skin cancer, unusual brain tumors, skeletal abnormalities (such as bifid ribs), and macrocephaly	[15]
Carney complex	Autosomal dominant	PRKAR1A	Myxoma	5%	Endocrine overactivity, with Cushing’s syndrome or acromegaly, and spotty skin pig-mentation (i.e., lentiginosis)	[16]

A subset of cardiac tumors is associated with specific genetic syndromes, and therefore the patients may present with additional extracardiac signs and symptoms, described in the column on the right. (TSC = tuberous sclerosis complex; PTCH1 = patched 1 gene; PRKAR1A = cAMP dependent protein kinase type 1α regulatory subunit).

**Table 2:** Case reports of tumoral phenocopies and main features of cardiac tumors.

Tumor	Typical Location	Histologic Features	CMR Features	Notes	Case Reports in Literature
**Benign congenital**					
Rhabdomyoma	Ventricle	Large and vacuolated myocytes (spider cell)	T1 isointense, T2 iso/hyperintense, no/minimal contrast enhancement	Strongly (80%) associated with tuberous sclerosis	[17]
Hamartoma of mature cardiac myocytes	Ventricle	Enlarged, highly disorganized cardiomyocytes	T1 Isointense T2 inhomogeneously hyperintense	Very similar to HCM	[18,19]
Cardiac fibroma	Ventricle and ventricular septum	Tumor cells resemble fibroblasts, calcification is common	T1 and T2 hypointense, heterogeneous late contrast enhancement with hypoenhancing core	Found in 3% of patients with Gorlin syndrome	[20,21,22,23]
**Benign acquired**					
Myxoma	Left atrium, right atrium, interatrial septum	Splinde-shaped cells (myxoma cells), calcification can be present	T1 iso/hypointense, T2 hyperintense, heterogeneous late contrast enhancement of >50% of the mass area	5% are a manifestation of the Carney complex. In case of haemorrhage and haemosiderin deposition, hypointense in all sequences	[24]
Hemangioma	Left atrium, right atrium	Variably sized blood vessels	T1 iso/hyperintense, T2 hyperintense, peripheral nodular contrast enhancement and progressive centripetal fill	Sometimes with heterogeneous enhancement due to calcifications and septations of the mass	[25]
Lipomatous Hypertrophy	Interatrial septum	Brown fat and cardiac myocytes	T1 and T2 hyperintese, no contrast enhancement	Intensity reduced with fat suppression technique	[26,27,28]
**Malignant**					
Lymphoma	Right atrium	Malignant lymphocytes	T1 and T2 isointense, heterogeneous enhancement	Highly abnormal T2 relaxation times (up to 140 ms) with T2 -mapping	[29,30,31,32,33,34,35]
Metastases	Variable	Infiltrating malignant cells	Heterogeneous enhancement		[36,37,38,39]
Metastases of melanoma	Variable	Infiltrating malignant cells	T1 hyperintense, T2 hypointense	Due to the T1 relaxation time-shortening properties of melanin	[40,41]

Cardiac tumors mimicking HCM may be benign or malignant. Their recognition can be based upon their histological and CMR features. The column on the far right refers to the publications describing tumoral phenocopies. In line with what stated above, malignant cardiac tumors seem to be the most frequent tumoral phenocopies. (CMR = cardiac magnetic resonance; HCM = hypertrophic cardiomyopathy).

**Table 3:** Case reports describing a tumoral phenocopy of hypertrophic cardiomyopathy (HCM) in which cardiac magnetic resonance (CMR) was performed.

Tumoral Phenocopy of HCM	Patient		Symptoms	ECG Suggestive	Echocardiography	CMR Depiction		Notes	Reference
	Age	Gender			Mass	Mass	Diagnostic		
Rhabdomyoma	50	Male	Yes	No	No	Yes	Yes		[17]
Hamartoma of mature cardiac myocytes	33	Male	Yes	NR	Not at first evaluation	Yes	No	Definitive diagnosis obtained with histology of the tumor excised	[18]
Hamartoma of mature cardiac myocytes	41	Female	Yes	Yes	Not at first evaluation	Yes	No	Definitive diagnosis obtained with histology of biopsy	[19]
Cardiac fibroma	55	Male	Yes	Yes	Not at first evaluation	Yes	Yes	Precedent misdiagnosis of HCM	[21]
Cardiac fibroma	40	Male	No	Yes	Inadequate window	Yes	Yes		[20]
Cardiac fibroma	59	Female	Yes	Yes	No	Yes	Yes		[23]
Myxoma	5	Male	No	Yes	Not at first evaluation	Yes	Yes		[24]
Cardiac angioma	20	Female	Yes	Yes	No	No	No	Precedent misdiagnosis of HCM; definitive diagnosis obtained with histology of septal myectomy sample	[25]
Lipomatous hypertrophy of interventricular septum	46	Male	No	No	No	Yes	Yes		[26]
Lipomatous hypertrophy of interventricular septum	17	Male	No	No	No	Yes	Yes		[27]
Non-Hodgkin Lymphoma	38	Male	No	NR	No	No	Yes	At CMR myocardial involvement compatible with lymphoproliferative disease	[29]
Burkitt’s Lymphoma	34	Female	No	Yes	No	No	No	Concomitant oncological history; definitive diagnosis with 18-FDG PET and regression after chemotheraphy	[30]
Metastases Melanoma	45	Female	No	NR	NR	Yes	Yes	Known history of malignant melanoma	[40]
Metastases Hepatocellular Carcinoma	61	Male	Yes	Yes	Not at first evaluation	Yes	No	Previous diagnosis of HCC and misdiagnosis of HCM; myocardial biopsy needed for conclusive diagnosis	[36]

In most of the cases, the complementary use of CMR was necessary to detect the cardiac tumor and support the final diagnosis. However, in a subset of cases, the final diagnosis was obtained only with histological analysis. (HCM = hypertrophic cardiomyopathy; ECG = electrocardiogram; CMR = cardiac magnetic resonance; NR = not reported; 18-FDG PET = 18-fluorodeoxyglucose positron emission tomography; HCC = hepatocellular carcinoma).

**Table 4:** Red flags suggesting a possible tumoral phenocopy of HCM.

Red Flag	Description	Rationale
Clinical	Prior clinical history of malignancy	Secondary cardiac metastases can occur many years after the diagnosis of a primary tumor
	Extracardiac syndromic signs and symptoms	A subset of cardiac tumors is associated to genetic syndromes: Tuberous sclerosis; Gorlin syndrome; Carney complex.
Echocardiografic	Concomitant hypertrophy of RV, atria, or IAS	Their presence imply a higher probability of a phenocopy (tumors, amyloid or Fabry disease) and make the diagnosis of HCM less likely
	Atypical pattern of LVH	
	Pericardial and/or pleural effusion at least moderate	
	Absence of ancillary findings of HCM	Tumoral phenocopies of HCM described never show the ancillary echocardiographic findings often associated with typical HCM (anomalies of papillary muscles, elongated mitral valve leaflets, myocardial clefts).

The presence of red flags warrants CMR in addition to echocardiography in order to avoid missing a tumoral phenocopy. (HCM = hypertrophic cardiomyopathy; LVH = left ventricular hypertrophy; RV = right ventricle; IAS = interatrial septum; CMR = cardiac magnetic resonance; Dx = diagnosis).

## Abbreviations

HCM	Hypertrophic cardiomyopathy
LV	Left ventricle
LVOTO	Left ventricular outflow tract obstruction
CMR	Cardiac magnetic resonance
HCC	Hepatocellular carcinoma
TSC1	Tuberous sclerosis complex 1
TSC2	Tuberous sclerosis complex 2
PTCH1	Patched 1 gene
AHCM	Apical HCMECV = Extracellular volume
LGE	Late gadolinium enhancement
STIR	Short-tau inversion recovery
SSFP	steady-state free precession
LVH	LV hypertrophy
ECG	Electrocardiogram
OHCM	Obstructive HCM
SAM	Systolic anterior motion
CT	Computed tomography
ICD	Implanted cardioverter-defibrillator
SCD	Sudden cardiac death
ESC	European Society of Cardiology
AHA/ACC	American Heart Association/American College of Cardiology
SCMR	Society for Cardiovascular Magnetic Resonance
